# Machine Learning Model for Imbalanced Cholera Dataset in Tanzania

**DOI:** 10.1155/2019/9397578

**Published:** 2019-07-25

**Authors:** Judith Leo, Edith Luhanga, Kisangiri Michael

**Affiliations:** Nelson Mandela African Institution of Science and Technology (NM-AIST), School of Computation and Communication Science and Engineering (CoCSE), P.O. BOX 447, Arusha, Tanzania

## Abstract

Cholera epidemic remains a public threat throughout history, affecting vulnerable population living with unreliable water and substandard sanitary conditions. Various studies have observed that the occurrence of cholera has strong linkage with environmental factors such as climate change and geographical location. Climate change has been strongly linked to the seasonal occurrence and widespread of cholera through the creation of weather patterns that favor the disease's transmission, infection, and the growth of* Vibrio cholerae*, which cause the disease. Over the past decades, there have been great achievements in developing epidemic models for the proper prediction of cholera. However, the integration of weather variables and use of machine learning techniques have not been explicitly deployed in modeling cholera epidemics in Tanzania due to the challenges that come with its datasets such as imbalanced data and missing information. This paper explores the use of machine learning techniques to model cholera epidemics with linkage to seasonal weather changes while overcoming the data imbalance problem. Adaptive Synthetic Sampling Approach (ADASYN) and Principal Component Analysis (PCA) were used to the restore sampling balance and dimensional of the dataset. In addition, sensitivity, specificity, and balanced-accuracy metrics were used to evaluate the performance of the seven models. Based on the results of the Wilcoxon sign-rank test and features of the models, XGBoost classifier was selected to be the best model for the study. Overall results improved our understanding of the significant roles of machine learning strategies in health-care data. However, the study could not be treated as a time series problem due to the data collection bias. The study recommends a review of health-care systems in order to facilitate quality data collection and deployment of machine learning techniques.

## 1. Introduction

Cholera is an acute epidemic infectious disease caused by* Vibrio cholerae (V. cholerae) *bacteria [1]. The bacteria typically live in waters which are salty and warm, such as estuaries and water along with the coastal areas. People contract* V. cholerae *after drinking liquids or eating foods contaminated with the bacteria [2]. The disease remains to be notorious and a threat to human society throughout history, due to the extraordinary scale of death and damage it brought over the years [3].

### 1.1. Historical Background of Cholera Disease

At first, the root of cholera was unknown so it caused devastating mortality of millions of people across the globe and thus contributed to massive panic to countries where it appeared [4]. According to the literature, there have been a total of seven cholera pandemics [5]. Cholera pandemic is a cholera epidemic that can last many years or even a few decades at a time and that spreads to many countries and across continents and oceans [6]. The first cholera pandemic occurred from 1817 to 1824 in India and spread to Southeast Asia, Central Asia, the Middle East, China, and Russia, leaving hundreds and thousands of people dead [7]. The second cholera pandemic occurred in 1826 to 1837 in India and spread to western Asia, Europe, Great Britain, and the Americas, as well as east of China and Japan. It caused more deaths, more quickly than any other epidemic disease in the 19th century [8]. The third cholera pandemic also caused the highest fatalities in the 19th century [9]. It originated in India and spread far beyond its borders to Russia and Great Britain. Researchers at the University of California, Los Angeles, believe that the third cholera pandemic started as early as 1837 and lasted until 1863. From 1853 to 1854, the pandemic caused 23,000 deaths in Great Britain and over 10,000 deaths in London. As the results of the August 1854 cholera outbreak in London, John Snow identified contaminated water as the means of transmission of the disease. He mapped a cluster of cholera cases near a water pump in one neighborhood. His breakthrough led to the control of cholera epidemics in the 19th century [9].

**Figure 1:**
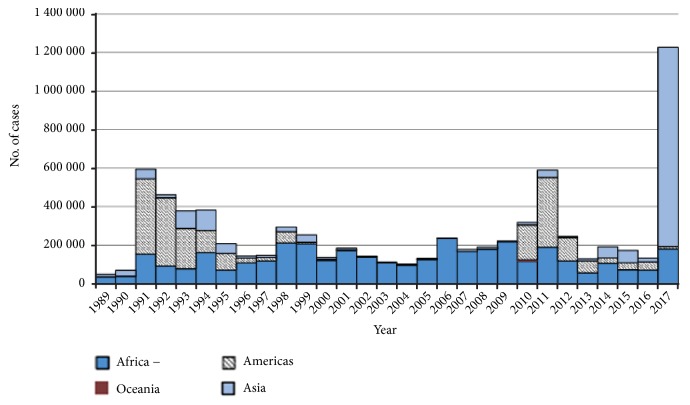
Cholera cases reported by WHO by a year and by continent from 1989 to 2017 [43].

However, there were other cholera pandemics after John Snow's breakthrough, such as the fourth cholera pandemic which began in 1863 and ended in 1875, the fifth cholera pandemic (1881 to 1896), the sixth cholera pandemic (1899 to 1923), and the seventh cholera pandemic (1961 to the 1970s) [6, 10]. During the fourth pandemic, cholera spread throughout the Middle East and was carried to Russia, Europe, and North America and reached North Africa where it spread to Sub-Saharan Africa (SSA), killing 70,000 in Zanzibar, Tanzania in 1869 [11]. To date, cholera is still prevalent in SSA areas with inadequate sanitation, poor food, and water hygiene and remains a major global public health problem [12], as indicated in Figure 1.

### 1.2. Transmission and Infection

Cholera disease is usually transmitted through the fecal-oral route of contaminated food or water caused by poor sanitation [13]. Most cholera cases in developed countries are transmitted through contaminated food, whereas, in developing countries, it is more often through contaminated water [3]. Food transmission can occur when people harvest seafood such as oysters and shellfish in the waters infected with* V. cholerae. *People infected with cholera often have diarrhea and hence disease transmission may occur if this diarrhea contaminates water used by other people [14]. A single diarrheal incident can cause a one million increase in numbers of* V. cholerae *in the environment through waterways, groundwater, and drinking water supplies. Normally, the transmission of cholera directly from person to person is very rare [15]. 

*V. cholerae* can also exist outside the human body in natural water sources, either by itself or through contracting with phytoplankton, zooplankton, and biotic and abiotic detritus. Hence, drinking such water can also result in cholera disease, even without prior contamination through fecal matter [16]. In addition, there are several virulence factors which can easily contribute to the pathogenicity of the* V. cholerae* to easily infect and cause symptoms to the hosts [17]. These virulence factors include toxin coregulated pilus, cholera toxin, and motility [18]. Furthermore, in our rapidly changing environment, it has been reported by several researchers that the transmission and infection of cholera epidemics are greatly influenced by seasonal weather variation [19]. This is because the dynamics of weather patterns dictate the infection and transmission rate of cholera disease. As they affect natural demographic behavior of population involved and also influences almost all variables involved in the growth of* V. cholerae. *Moreover, the fluctuation of weather variables, such as temperature, rainfall, humidity, and wind, is also regarded as the core factor that causes reemergence of cholera outbreak cycles and its variability from small to large scales [20].

### 1.3. Foundation of Machine Learning

Recently, the global climatic change has led to the massive fluctuation of seasonal weather changes and environmental conditions [21], which has resulted in rapid cholera outbreaks in the world, especially in the developing countries [22], such as Tanzania, Nigeria, Zimbabwe, and Malawi [23, 24]. In addition, it has been noted that the global burden of cholera epidemics from the seasonal weather changes and environmental factors is expected to increase over time with a rapid increase of epidemic size [25, 26]. With the limited number of the workforce in the Tanzanian health-sector and the use of manual mechanisms [27] henceforth, there is a dire need to develop a suitable cholera prediction model for early warning mechanisms [28, 29]. Over the past decades, there have been several studies and great achievements in developing epidemic models and systems for the proper prediction of cholera. However, the integration of weather variables and the use of machine learning techniques have not been deployed in modeling the cholera epidemics in Tanzania's settings [30]. This is due to the challenges that come with its datasets such as imbalanced data, missing information, and other uncertainties [31]. Machine learning is an application of artificial intelligence that provides computer-based systems with the ability to automatically learn and improve from experience without being explicitly programmed [32]. Machine learning is categorized mostly into supervised and unsupervised algorithms. Supervised algorithms are used when the data used to train is classified and labeled while unsupervised algorithms are used in unlabeled data [33, 34]. The basic premise of machine learning is to build models that can receive input data and use statistical analysis to predict an output while updating outputs as new data becomes available. [35]. Over the past years, data determined extensively the success of machine learning algorithms; however, with the introduction of innovative strategies such as sampling, decomposition, scaling, and aggregation, there has been great revolution [36]. Nowadays, machine learning is used in a wide range of applications such as timely decision making, virtual personal assistance, social media services, video surveillance, identifying disease and diagnosis, drug discovery, and clinical researches, since it is capable of handling data innovatively towards achieving its intended goals [37, 38].

In addition, with the current growing number of data in the health sectors due to the availability of cost-effective mechanisms for collecting and storing health-care data, other techniques such as traditional statistical techniques are losing power [39]. This is because nowadays machines can handle a large amount of data in terms of online storage and low-cost computation and processing without the need to reduce them through the use of mathematical techniques [40]. Hence, this breakthrough has given power to the rise of machine learning techniques [41]. This paper, therefore, proposes the use of machine learning techniques to model cholera epidemics with linkage to seasonal weather changes while overcoming the data imbalance problem in Tanzania. This is because machine learning techniques are believed to be very powerful, advanced, and innovative tools for studying the dynamics of epidemics with a wide range of dynamic and complex variables such as seasonal weather variability and imbalanced dataset condition [42]. The rest of this paper includes Section 2 which provides the materials and methods, Section 3 which presents the results and brief discussion, and, lastly, Section 4 which concludes with a brief discussion.

## 2. Methodology

This work used design science research methodology (DSRM) [44] to formulate the cholera model with its linkage to seasonal weather changes. The methodology consists of six steps iterations: problem identification and motivation, design and development, demonstration of the product, evaluation, and communication through publishing the results. In addition, DSRM is an outcome-based methodology which focuses on improving the functional performance of the artifacts such as algorithms [45].

### 2.1. Study Area

Dar es Salaam region in Tanzania was chosen to be our study area. This is because Tanzania is a developing country in SSA with frequent reemergence of cholera epidemics [46]. Most of the cholera outbreaks in Tanzania are believed to start from Dar es Salaam region and spread throughout to other regions such as Kigoma, Morogoro, and Tanga, except for a few cases of the cholera outbreak which happened in Kigoma in 2015 due to the overcrowded crisis of Burundi refugees [47]. Researchers believed that the dynamics of cholera epidemics in Dar es Salaam are strongly linked to the weather variation [48]. This is because Dar es Salaam region is an industrial area, with the largest number of population compared to other regions in the country [49]. In addition, the region has limited resources to sustain peoples' daily needs and also has poor sanitary and hygiene conditions. Hence, the region becomes easily vulnerable to the rapid spread of the disease especially when favorable weather conditions are met such as heavy rainfall [50]. Furthermore, the country has only focused on the use of medical supplies such as water treatment chemicals instead of developing effective models or system for early prediction, and appropriate analysis of cholera epidemics [26].

### 2.2. Data

The data was collected in Dar es Salaam region from January 2015 to December 2017, which includes seasonal weather variables such as temperature, rainfall, humidity, and wind, from Tanzania Meteorological Agency (TMA), and cholera cases data which includes district-location of the patient, the date onset for cholera-patient diagnosis and patients' laboratory results, from the Ministry of Health and Social Welfare as shown in Tables 1 and 2.

The date onset variable was collected in order to assist the exercise of aligning the weather variables to the corresponding patient's details. In addition, the study considered the date onset as the date when a patient contracted* V. cholerae*. This is because the incubation period of* V. cholerae* is five days and also the range of weather variables within a week is always insignificant [51, 52].

### 2.3. Statistical Data Description

**Figure 2:**
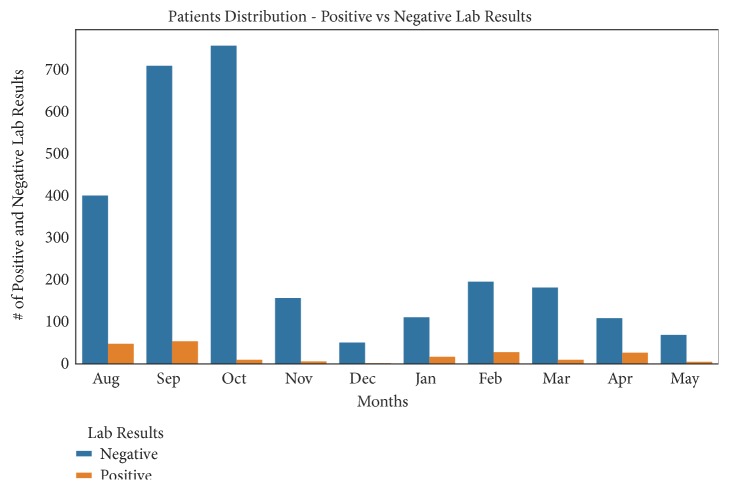
Patients distribution per months.

**Figure 3:**
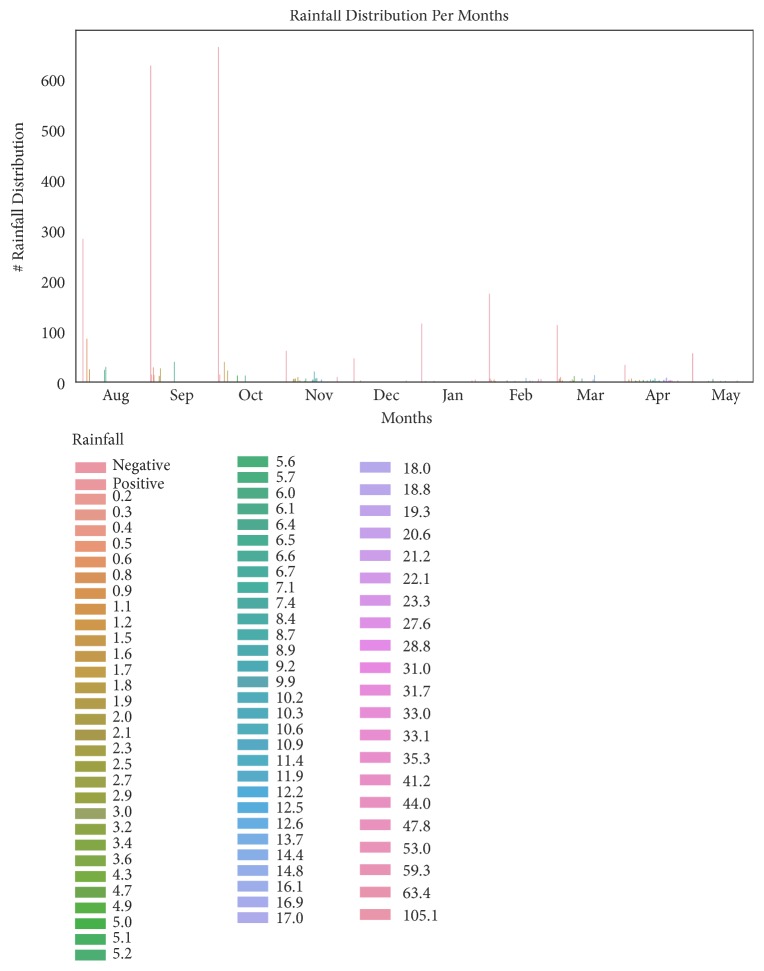
Rainfall distribution per months.

**Figure 4:**
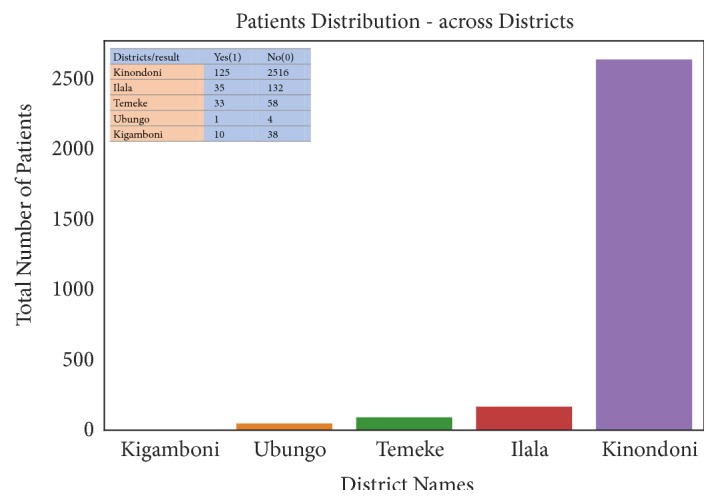
Patient distribution across districts.

This subsection describes data into statistical measures of counts, means, standard deviations (std), Minimum (min), Maximum (max), 25th, 50th, and 75th percentile, as shown in Table 3. In Table 3, the count shows the total number of collected data in each column, mean shows the mean value of each column, min and max show the minimum and the maximum number of each column respectively, and std shows the standard deviation of each column [54]. In addition, it summarizes the data into graphical representations as shown in Figures 2, 3, and 4. Whereby Figure 2 presents patients distribution per months, Figure 3 shows rainfall distribution per months and Figure 4 shows patients distribution across districts.

### 2.4. Data Preprocessing

Following the collection of data with 2951 patients and 9 predictors, the data collected was checked for the presence of error in data entry including missing data and misspellings. Following this process, there was no error in misspelling; however, there were 10 missing weather data. Hence, we had to visit TMA offices in order to cross-check the received data and fill the missing data. The complete data was stored in Microsoft Excel of Microsoft office 2013 suite of desktop publishing (.xls). Lastly, the data was transformed into the comma separated variable (.csv) file. Then, using python, we scaled features according to a minimum and maximum value (MinMaxScaler) between 0 and 1 in order to improve the distance-based approach in the dataset.

### 2.5. Model Formulation Approach

**Figure 5:**
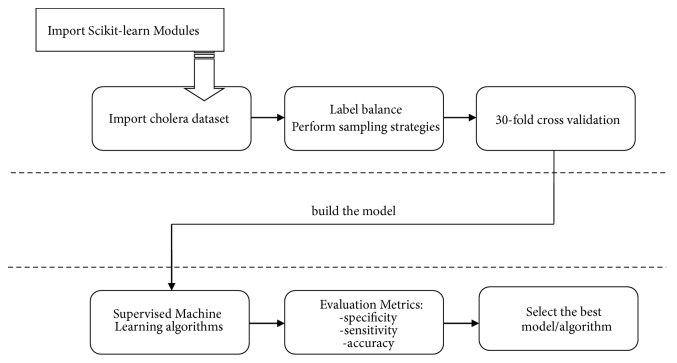
Model formulation approach.

In order to achieve our model, we followed the procedure as briefly explained in Figure 5. In this procedure, we first imported the scikit-learn modules then loaded the cholera datasets. After that, we checked how the dataset is balanced and performed sampling procedure in order to balance the dataset. Then we did 30-fold cross validation as a test method in order to reduce variability, overfitting, and selection bias [37]. Then, the training data was used to build the models and the testing data was used to assess the prediction performance of the models. Lastly, after building the model, we performed evaluation metrics in order to select the best performing models or algorithms.

### 2.6. Machine Learning Models

Based on the study, we used supervised machine learning algorithms because their main goal is to learn a target function that can be used to predict values of a class. In addition, supervised algorithms can easily map an input to an output [55]. In a nutshell, in machine learning, there is no one algorithm that works best for every problem since there are many factors at play such as the size and structure of the datasets. Therefore, we selected the best seven supervised machine learning algorithms which will be evaluated based on their performance. Then, the best algorithm will be selected to conduct this study. The following are the selected algorithms: XGBoost, K-Nearest Neighbors (K-NN), Decision Tree, Random Forest, ExtraTree, AdaBoost, and Linear Discriminant Analysis (LDA) [56]. The following is a brief discussion of their description.

XGBoost is an optimized distributed gradient boosting algorithm designed to be highly efficient, flexible, and portable. It provides a parallel tree boosting that solves many data science problems in a fast and accurate way [57], whereas Decision Tree is one of the most popular machine learning algorithms that use tree-like model decisions and their possible consequences. It is capable of fitting complex datasets while allowing the user to see how a decision was taken [58]. K-NN is a simple and nonparametric algorithm used for classification and regression. It is often successful in classification situation where the decision boundary is very irregular [59]. LDA is a well-established machine learning technique for predicting categories. It is frequently used as a dimensional reduction technique for pattern recognition or classification [60]. Random Forest is a meta-estimator which fits a number of decision trees on various subsamples of the dataset. Then, it averages the results in order to improve predictive accuracy and control overfitting [61]. An AdaBoost classifier is a short form for Adaptive Boosting meta-estimator that begins by fitting a classifier on the original dataset and then fits additional copies of the classifier on the same dataset. It is powerful and flexible and can be used in conjunction with many other types of learning algorithms in order to improve performance [38]. Lastly, ExtraTree classifier is a meta-estimator that fits a number of randomized decision trees on various subsamples of the dataset and uses averaging to improve the prediction accuracy and control overfitting [62].

### 2.7. Data Imbalance Problem

**Figure 6:**
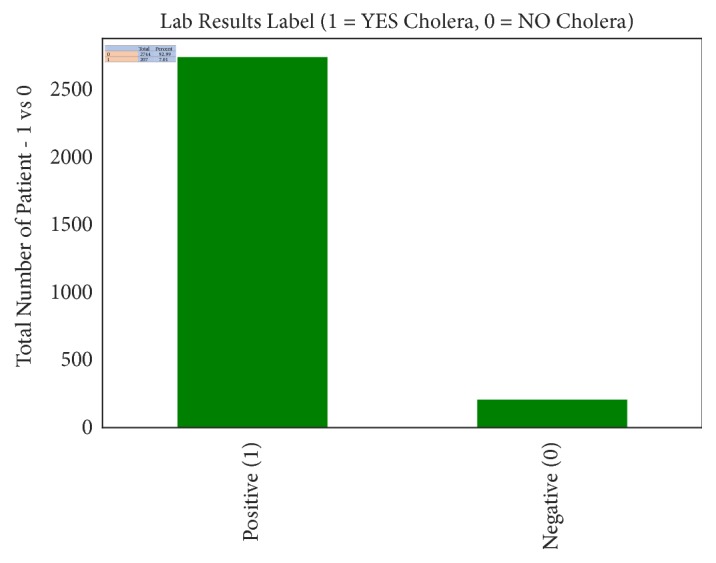
Imbalanced cholera dataset.

The collected dataset is imbalanced at a rate of 0.07 as shown in Figure 6. The data imbalance problem is one of the major challenges in the field of machine learning [63]. This is because most of the machine learning algorithms assume that dataset is equally distributed. In the case of this study, the majority class which is* NO Cholera (0)* has dominated the prediction value. Hence our prediction poorly classifies the observation of the minority class, which is* YES Cholera (1)*. We performed oversampling by using Adaptive Synthetic Sampling Approach (ADASYN), which is an improved version of Synthetic Minority Oversampling Technique (SMOTE) in order to restore sampling balance. ADASYN was selected because it can easily reduce the learning bias introduced by the original imbalance data distribution and also it adaptively shifts the decision boundary towards the difficulty to learn samples. In addition, ADASYN is independent of underlying classifier and can be easily implemented [64]. Furthermore, we also performed decomposition or dimensional reduction of the dataset with Principal Component Analysis (PCA). PCA reduces the high dimensionality of data by selecting an optimal feature from the original dataset [65].

### 2.8. Model Evaluation Metrics

At this stage, we evaluated the seven best supervised machine learning algorithms in order to select the best fit model. Based on the nature of cholera dataset, we used balanced-accuracy, sensitivity, and specificity metrics to evaluate the performance of the models as shown in Table 5. The balanced accuracy was performed on the dataset so that the noncholera label is not overvalued due to the number of samples present. Specificity and sensitivity are metrics parameters that together define effectively the presence or absence of specific condition such as outbreak or diseases. Sensitivity is the ability of a test to correctly classify an individual as diseased, and specificity is the ability of a test to correctly classify an individual as disease-free.  Table 4 shows the relation between sensitivity and specificity.

### 2.9. Model Selection

Based on the evaluation metrics, we obtained two models with the best results. We then performed a statistical hypothesis test using the Wilcoxon sign-rank test in order to compare them and select one model for the study. The Wilcoxon sign-rank test is a nonparametric analysis that statistically compares the average of two dependent models and then assesses significant differences [66]. The test is very robust and efficient and does not depend on the parent distribution or parameters of the datasets. In addition, it does not require any assumption about the shape of the distribution to determine its results [67].

## 3. Results and Discussion

This section briefly presents the results and their discussion.

### 3.1. Result

**Figure 7:**
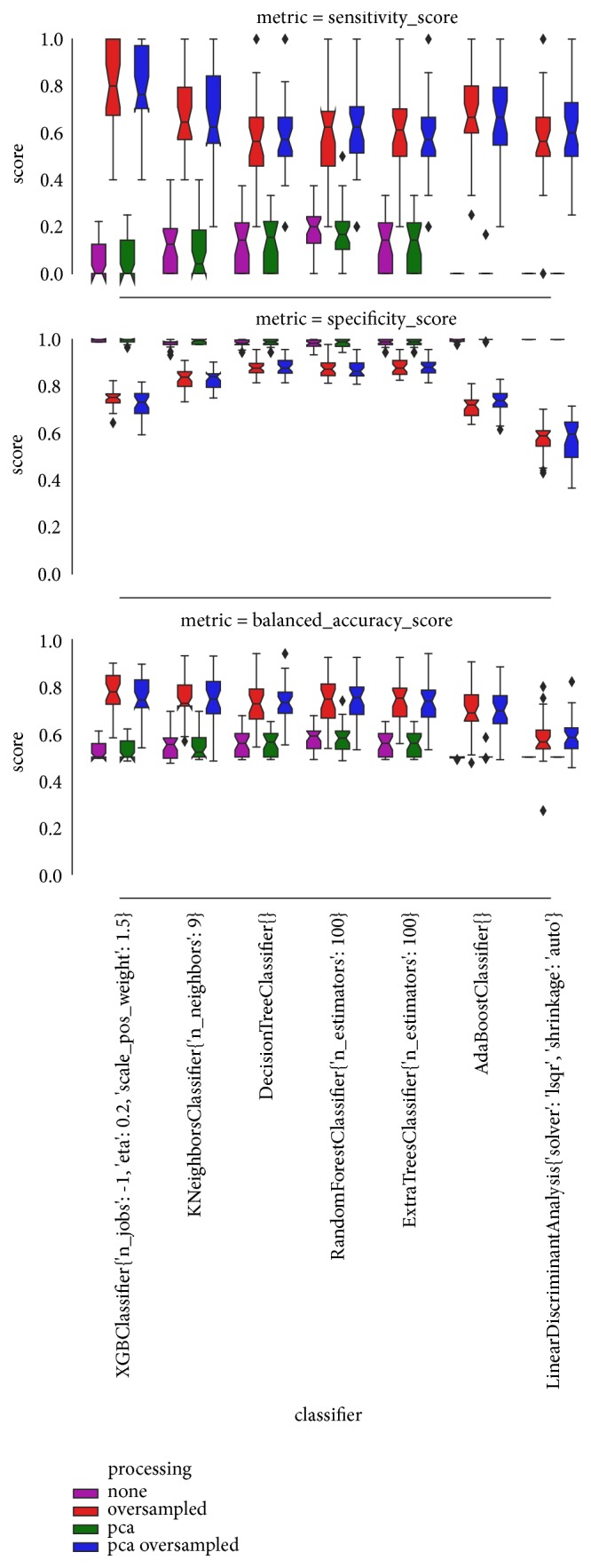
Results of sensitivity, specificity, and balanced-accuracy metrics.

Based on the obtained results, XGBoost and K-NN perform best with respect to the chosen metrics as shown in Figure 7.

### 3.2. Discussion

The success of machine learning in predicting cholera occurrence with linkage to the seasonal weather changes relies on the good use of data and machine learning classifier. Selecting the right machine learning model for the right problem is necessary for achieving the best results. The results from Figure 2 and Table 3 show clearly that the K-NN and XGBoost algorithms perform well compared to the other five algorithms in terms of their sensitivity, specificity, and balanced-accuracy metrics. However, after performing the Wilcoxon sign-rank test between K-NN and XGBoost algorithms, there is insufficient evidence to differentiate the results of their metric. Based on the main objective of the study and features of the two algorithms, the XGBoost classifier was selected to be the best model for this study. This is because XGBoost algorithm is an implementation of gradient boosted decision trees designed to be highly efficient, flexible, and portable and has the ability to increase execution speed and model performance. It is greatly applicable in anomaly detection of supervised settings where data is often highly imbalanced such as DNA sequencing, credit card transactions, and cybersecurity. In addition, the XGBoosting algorithm has the parameter “scale-pos-weight” to focus on the sensitivity of the data and also provides step by step strategy to deal with imbalanced datasets. Furthermore, XGBoost is useful in decision making since it embeds decision trees in its procedures; therefore, it aligns with the main goal of the study [57], whereas K-NN does not work well with large datasets, data with nonhomogeneous features, high dimensional, and imbalanced conditions. In addition, K-NN has no capability of dealing with missing value problems and its accuracy can be severely degraded by the presence of irrelevant features [59, 68]. With these brief details of the two models, XGBoost classifier was selected to be the best model for this study.

Furthermore, the result of data analysis indicates that there are a larger number of patients with cholera in August, September, and April than the other months. In addition, the temperature ranges from 22°C to 32°C, rainfall level is greater than 50 mm, and humidity level is greater than 75% favoring the occurrence of cholera incidences. Furthermore, based on feature importance analysis, temperature mean ranked number one, followed by rainfall, then humidity, wind speed and lastly wind direction. Moreover, the study could not be treated as a time series problem due to the poor quality of data and data collection bias. Nearly, all data is from Kinondoni district and few entries are from the other four districts. This is due to poor data collection especially in Kigamboni, Ubungo, and Temeke districts [69, 70]. In addition, the independent variables do not include proper time information; therefore, the model is unable to leverage time features and cannot do fair predictions. However, with these limitations, the selected model is useful in predicting accurately cholera epidemics using future weather variables. Furthermore, the K-NN model can handle the existing large amount of data in our health sectors, reduce computational performance, and also produce timely and reliable results for early decision making [40, 71]. Lastly, the study has significantly improved our understanding of how we can improve in the health-care systems and policies in Tanzania. Future work is to rerun the model with new weather datasets in order to predict cholera cases.

## 4. Conclusion

The transmission of cholera epidemics occurs in various pathways which makes its modeling very challenging. Looking further at the challenge that most of the collected cholera datasets bring such as imbalanced data, missing information and dynamic nature of its predictors such as weather variability, it becomes more difficult to formulate the suitable model. In this study, we managed to model cholera epidemics linked with weather variables. The study improved our understanding of how imbalanced dataset should be treated towards mitigating the prediction performance of the models, and the role of oversampling and machine learning strategies in health-care data. As a result, the XGBoost machine learning algorithm was selected to be the best cholera predictor based on the used dataset. The study recommends a review of health-care systems in order to facilitate quality data collection and deployment machine learning techniques, which will significantly manage the complexity of real-world problems such as data-driven analysis, decision making, prediction and eradication strategies of cholera epidemics at large scale.

## Figures and Tables

**Table 1:** Description of data for daily seasonal weather changes.

Variable	Description	SI Unit
Temp_max	Minimum Temperature	Degree centigrade (°C)
Temp_mean	Mean Temperature	Degree centigrade (°C)
Temp_min	Maximum Temperature	Degree centigrade (°C)
Temp_range	Temperature Range	Degree centigrade (°C)
Rainfall	Rainfall	Millimeter (mm)
Humidity	Relative Humidity	(%)
Wind_Spd	Wind Speed	Knots
Wind_Dir	Wind Direction	Degrees

**Table 2:** Description of cholera cases data with regards to patient details.

Variable	Description	SI Unit
District	District Names	Dar es Salaam Districts
Date	Date on set	Date Month Year
Result	Lab result	Yes or No

**Table 3:** Statistical data description of cholera cases using count, mean, std, min, max, and percentile.

	count	mean	std	min	25%	50%	75%	max
Rainfall	2951	1.962	7.518	0	0	0	0.2	105.1
Temp_max	2951	31.343	1.816	0	30	31.1	32.7	36.3
Temp_min	2951	22.496	2.505	0	21	21.4	24.2	28.8
Temp_mean	2951	26.92	1.854	0	25.5	26.7	28.2	31.55
Temp_range	2951	8.847	2.323	0	7.5	9	10.4	16.4
Humidity	2951	78.835	5.2	0	75	78	81	97
Wind_Dir	2951	117.32	91.23	0	50	120	160	360
Wind_Spd	2951	5.33	3.706	0	3	5	8	18
result	2951	0.07	0.255	0	0	0	0	1

**Table 4:** Description of sensitivity and specificity [53].

	Disease present	Disease absent	Total
Test positive	a (TP)	b (FP)	all cases

Test negative	c (FN)	d (TN)	all noncases

	all diseased	all nondiseased	all participants in the study

	Sensitivity= a/(a+c)	Specificity= d/(b+d)	

*Note:* TP: True Positive, TN: True Negative, FP: False Positive, and FN: False Negative.

**Table 5:** Classifiers with a detailed range of sensitivity, specificity, and balanced accuracy.

Classifiers	Sensitivity score	Specificity score	Balanced accuracy score
*Plain Classifiers *			
XGB	0.055+-0.08	0.995+-0.006	0.525+-0.04
K-NN	0.095+-0.103	0.985+-0.014	0.54+-0.053
DT	0.119+-0.116	0.98+-0.016	0.549+-0.061
RF	0.166+-0.137	0.981+-0.016	0.574+-0.072
ExtraTrees	0.114+-0.113	0.984+-0.015	0.549+-0.06
AdaBoost	0	0.997+-0.005	0.498+-0.003
LDA	0	1	0.5

*Oversampling Classifiers*			
XGB	0.801+-0.148	0.742+-0.053	0.772+-0.079
K-NN	0.656+-0.24	0.83+-0.042	0.743+-0.123
DT	0.579+-0.17	0.882+-0.032	0.73+-0.09
RF	0.632+-0.156	0.88+-0.034	0.756+-0.085
ExtraTrees	0.589+-0.161	0.88+-0.032	0.734+-0.085
AdaBoost	0.708+-0.206	0.707+-0.058	0.707+-0.103
LDA	0.593+-0.23	0.594+-0.051	0.593+-0.111

*PCA Classifiers*			
XGB	0.056+-0.096	0.9912+-0.01	0.524+-0.049
K-NN	0.061+-0.09	0.989+-0.013	0.525+-0.045
DT	0.119+-0.117	0.983+-0.015	0.551+-0.062
RF	0.153+-0.121	0.983+-0.016	0.568+-0.063
ExtraTrees	0.114+-0.113	0.984+-0.015	0.549+-0.06
AdaBoost	0	0.999+-0.004	0.5
LDA	0	1	0.5

*PCA/Oversampling Classifiers*			
XGB	0.805+-0.169	0.73+-0.05	0.767+-0.09
K-NN	0.705+-0.199	0.828+-0.034	0.767+-0.105
DT	0.596+-0.163	0.879+-0.032	0.737+-0.086
RF	0.645+-0.155	0.877+-0.031	0.761+-0.082
ExtraTrees	0.585+-0.19	0.879654+-0.033950	0.732+-0.102
AdaBoost	0.691+-0.168	0.731+-0.040	0.711+-0.087
LDA	0.534+-0.226	0.622+-0.072	0.578+-0.117

## Data Availability

The data used to support the findings of this study are available from the corresponding author upon request.
